# Older Adults and Technology Use: A Systematic Literature Review

**DOI:** 10.1093/geroni/igaa057.3004

**Published:** 2020-12-16

**Authors:** Hyung Wook Choi, Rose Ann DiMaria-Ghalili, Mat Kelly, Alexander Poole, Erjia Yan, Jina Huh-Yoo

**Affiliations:** 1 Drexel University, Philadelphia, Pennsylvania, United States; 2 Drexel University, Jenkintown, Pennsylvania, United States; 3 Information Science, Drexel University, Philadelphia, Pennsylvania, United States

## Abstract

Researchers are increasingly interested in leveraging technology to support the physical and mental well-being of older adults. We systematically reviewed previous scholars’ criteria for sampling older adult populations, focusing on age cohorts (namely adults over 65) and their use of internet and smart technologies. We iteratively developed keyword combinations that represent older adults and technology from the retrieved literature. Between 2011 and 2020, 70 systematic reviews were identified, 26 of which met our inclusion criteria for full review. Most important, not one of the 26 papers used a sample population classification more fine-grained than “65 and older.” A knowledge gap thus exists; researchers lack a nuanced understanding of differences within this extraordinarily broad age-range. Demographics that we propose to analyze empirically include not only finer measures of age (e.g., 65-70 or 71-75, as opposed to “65 and older”), but also those age groups’ attitudes toward and capacity for technology use.

